# Genomic analysis of *Salmonella* isolated from canal water in Bangkok, Thailand

**DOI:** 10.1128/spectrum.04216-23

**Published:** 2024-04-02

**Authors:** Jirachaya Toyting, Narong Nuanmuang, Fuangfa Utrarachkij, Neunghatai Supha, Yuwanda Thongpanich, Pimlapas Leekitcharoenphon, Frank M. Aarestrup, Toyotaka Sato, Jeewan Thapa, Chie Nakajima, Yasuhiko Suzuki

**Affiliations:** 1 Division of Bioresources, Hokkaido University International Institute for Zoonosis Control, Sapporo, Japan; 2 Research Group for Genomic Epidemiology, National Food Institute, Technical University of Denmark, Kgs. Lyngby, Denmark; 3 Department of Microbiology, Faculty of Public Health, Mahidol University, Bangkok, Thailand; 4 Laboratory of Veterinary Hygiene, Faculty of Veterinary Medicine, Hokkaido University, Sapporo, Japan; 5 Graduate School of Infectious Diseases, Hokkaido University, Sapporo, Japan; 6 One Health Research Center, Hokkaido University, Sapporo, Japan; 7 International Collaboration Unit, Hokkaido University International Institute for Zoonosis Control, Sapporo, Japan; 8 Division of Research Support, Hokkaido University Institute for Vaccine Research & Development, Sapporo, Japan; Universidad Andres Bello, Santiago, Chile

**Keywords:** *Salmonella*, canal, waterborne pathogens, genome analysis, drug resistance mechanisms

## Abstract

Antimicrobial resistance (AMR) poses an escalating global public health threat. Canals are essential in Thailand, including the capital city, Bangkok, as agricultural and daily water sources. However, the characteristic and antimicrobial-resistance properties of the bacteria in the urban canals have never been elucidated. This study employed whole genome sequencing to characterize 30 genomes of a causal pathogenic bacteria, *Salmonella enterica,* isolated from Bangkok canal water between 2016 and 2020. The dominant serotype was *Salmonella* Agona. In total, 35 AMR genes and 30 chromosomal-mediated gene mutations were identified, in which 21 strains carried both acquired genes and mutations associated with fluoroquinolone resistance. Virulence factors associated with invasion, adhesion, and survival during infection were detected in all study strains. 75.9% of the study stains were multidrug-resistant and all the strains harbored the necessary virulence factors associated with salmonellosis. One strain carried 20 resistance genes, including *mcr-3.1*, mutations in GyrA, ParC, and ParE, and typhoid toxin-associated genes. Fifteen plasmid replicon types were detected, with Col(pHAD28) being the most common type. Comparative analysis of nine *S*. Agona from Bangkok and 167 from public databases revealed that specific clonal lineages of *S*. Agona might have been circulating between canal water and food sources in Thailand and globally. These findings provide insight into potential pathogens in the aquatic ecosystem and support the inclusion of environmental samples into comprehensive AMR surveillance initiatives as part of a One Health approach. This approach aids in comprehending the rise and dissemination of AMR and devising sustainable intervention strategies.

**IMPORTANCE:**

Bangkok is the capital city of Thailand and home to a large canal network that serves the city in various ways. The presence of pathogenic and antimicrobial-resistant *Salmonella* is alarming and poses a significant public health risk. The present study is the first characterization of the genomic of *Salmonella* strains from Bangkok canal water. Twenty-two of 29 strains (75.9%) were multidrug-resistant *Salmonella* and all the strains carried essential virulence factors for pathogenesis. Various plasmid types were identified in these strains, potentially facilitating the horizontal transfer of AMR genes. Additional investigations indicated a potential circulation of *S*. Agona between canal water and food sources in Thailand. The current study underscores the role of environmental water in an urban city as a reservoir of pathogens and these data obtained can serve as a basis for public health risk assessment and help shape intervention strategies to combat AMR challenges in Thailand.

## INTRODUCTION

Antimicrobial resistance (AMR) is a growing global public health concern due to the widely used antimicrobial agent classes for both therapeutic and nontherapeutic purposes in humans, farm animals, aquaculture, and agriculture. The aquatic environment, such as rivers and canals, should play a significant role in disseminating AMR in society (1–3). However, surveillance of AMR in the aquatic environment is often overlooked compared with that of human clinical settings and has not been fully understood in many countries despite its importance (4–6).

*Salmonella* spp. is one of the causative pathogens of acute gastroenteritis, in which nontyphoid salmonellosis ranks among the top 10 leading causes of gastroenteritis in Southeast Asia, accounting for nearly 16 million cases and approximately 16,000 fatalities annually (7). While it was projected that 85% of salmonellosis cases resulted from foodborne infection, there is a potential risk of *Salmonella* infection from both contaminated drinking water and recreational water (7, 8). This is especially the case in warmer climates where the bacteria might survive and multiply in the environment. A previous study reported that *Salmonella* spp. was identified from 92.2% of Bangkok canal water samples (J. Toyting, N. Supha, Y. Thongpanich, J. Thapa, C. Nakajima, Y. Suzuki, and F. Utrarachkij, submitted for publication).

In the pathogenesis of salmonellosis, the contribution of several virulence factors is reported. The invasion of host cells and the ability to survive within them are crucial aspects of virulence. The invasion of host cells and intracellular survival rely on two distinct type III secretion systems (T3SS), namely, T3SS-1 and T3SS-2, each responsible for transporting specific sets of effector proteins. T3SS-1, flagella, fimbriae, and non-fimbrial adhesins regulate early invasion processes. Subsequently, activation of T3SS-2 and factors facilitating nutrient acquisition and antibacterial mechanism evasion support bacterial survival and replication in macrophages (9, 10). In addition, multidrug-resistant (MDR) *Salmonella* spp. has also been isolated from various water sources in some countries (11–13), suggesting that environmental water could potentially be a reservoir of *Salmonella* spp. with AMR.

Fluoroquinolones are one of the most frequently used antimicrobial agents for treating bacterial infections including salmonellosis. Because of their feature of chemical stability, these residues are often detected in wastewater that ultimately flows into surface waters (14), possibly leading to the emergence of fluoroquinolone-resistant bacteria. In addition to fluoroquinolones, β-lactams are among the top antibiotics consumed in humans and livestock. Notably, the ciprofloxacin and ceftriaxone resistance rates of nontyphoidal *Salmonella* in central Thailand have been increasing (15).

In Thailand, next-generation sequencing technology has been primarily employed in genome characterization of *Salmonella* spp. isolated from humans (16, 17), and pork and poultry production chains (18, 19) for identification of serotypes, sequence types (STs), virulence factors, antibiotic resistance genes (ARGs), and chromosomal-mediated gene mutations. On the other hand, implementation in characterizing *Salmonella* spp. from environmental sources remains notably scarce. Especially, characteristics and dissemination of the antimicrobial-resistant *Salmonella* spp. in the aquatic environment in the urban area have not been well elucidated. Bangkok, the capital city of Thailand, is home to over 11 million people and has an extensive canal network. The canal network is essential for their daily lives with diverse functions, encompassing drainage, wastewater and sewage management, transportation, agricultural resources, tourism, and recreational areas (20). This suggests that the molecular epidemiological analysis of *Salmonella* spp. in the canal of Bangkok provides beneficial scientific insights for understanding the dissemination of AMR through the aquatic environment based on public health and the One Health approach.

This study aimed to uncover the potential risk of AMR, virulence, and the contribution of urban canals as reservoirs by whole genome sequencing (WGS) of *Salmonella* strains derived from canal water samples in Bangkok. The data obtained from the present study should serve as a basis for public health risk assessment and suggest intervention strategies for AMR challenges in Thailand.

## RESULTS

### Species, serotypes, and ST types based on WGS

Species, serotypes, and ST types were summarized in Table 1. Twenty-nine of 30 strains were confirmed as *Salmonella enterica* subsp. *enterica*. One strain was identified as *Salmonella* sp. SJTUF14170, which is unclassified *Salmonella* and therefore was excluded from downstream analysis. Serotype prediction showed a high level of serotype diversity. The predominant serotype was Agona (31.0%, *n* = 9/29), followed by Mbandaka (10.3%, *n* = 3/29), and Stanley (10.3%, *n* = 3/29). The most common STs were identical to the serotype.

**TABLE 1:** Species, serotypes, and ST types of *Salmonella* spp. isolated from Bangkok canal water

Strain	Canal	Year	Species	Serotype	ST type
MU3	A	2016	*Salmonella enterica* subsp. *enterica*	Weltevreden	365
MU15	A	2016	*Salmonella enterica* subsp. *enterica*	Weltevreden	365
MU1	B	2016	*Salmonella enterica* subsp. *enterica*	Stanley	29
MU2	B	2016	*Salmonella enterica* subsp. *enterica*	Stanley	29
MU18	B	2016	*Salmonella enterica* subsp. *enterica*	Schwarzengrund	322
MU19	B	2017	*Salmonella enterica* subsp. *enterica*	Panama or Houston	48
MU4	B	2018	*Salmonella enterica* subsp. *enterica*	N/A	22
MU12	B	2018	*Salmonella enterica* subsp. *enterica*	Agona	13
MU7	B	2019	*Salmonella enterica* subsp. *enterica*	Agona	13
MU8	B	2019	*Salmonella enterica* subsp. *enterica*	Agona	13
MU17	B	2020	*Salmonella enterica* subsp. *enterica*	II 6,7:z29:[z42] or Tennessee	319
MU20	C	2017	*Salmonella enterica* subsp. *enterica*	Potential monophasic variant of Typhimurium (O5-)	34
MU23	C	2018	*Salmonella enterica* subsp. *enterica*	Stanley	29
MU5	C	2019	*Salmonella enterica* subsp. *enterica*	Give	516
MU9	C	2019	*Salmonella enterica* subsp. *enterica*	Agona	13
MU11	D	2016	*Salmonella enterica* subsp. *enterica*	Mbandaka	413
MU26	D	2016	*Salmonella enterica* subsp. *enterica*	Corvallis or Chailey	1541
MU21	D	2017	*Salmonella enterica* subsp. *enterica*	Mbandaka	413
MU22	D	2017	*Salmonella enterica* subsp. *enterica*	Agona	13
MU27	D	2017	*Salmonella* sp. SJTUF14170	-	-
MU13	D	2018	*Salmonella enterica* subsp. *enterica*	Farmsen or Poona	1069
MU28	D	2018	*Salmonella enterica* subsp. *enterica*	Corvallis or Chailey	1541
MU29	D	2019	*Salmonella enterica* subsp. *enterica*	Agona	13
MU14	D	2020	*Salmonella enterica* subsp. *enterica*	Kentucky	696
MU30	D	2020	*Salmonella enterica* subsp. *enterica*	Agona	13
MU6	E	2019	*Salmonella enterica* subsp. *enterica*	Give	516
MU25	E	2019	*Salmonella enterica* subsp. *enterica*	Apeyeme	1546
MU16	F	2019	*Salmonella enterica* subsp. *enterica*	Agona	13
MU24	F	2019	*Salmonella enterica* subsp. *enterica*	Agona	13
MU10	F	2020	*Salmonella enterica* subsp. *enterica*	Mbandaka	413

### AMR determinants in *Salmonella* strains from Bangkok canal water

**FIG 1:**
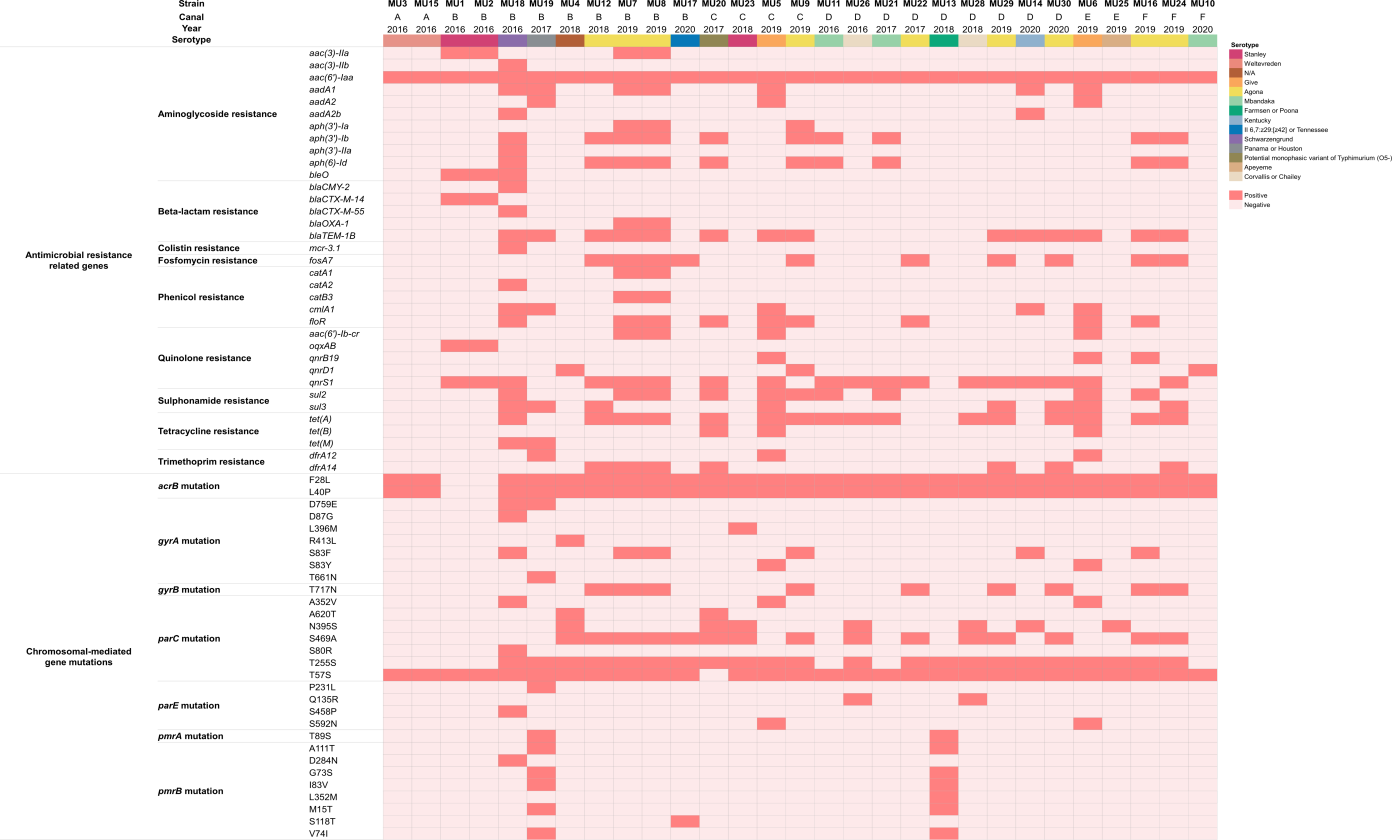
Heatmap of acquired AMR-related genes and chromosomal-mediated gene mutations in 29 *Salmonella* spp. isolated from Bangkok canal water. Present genes and gene mutations are shown in red and absent genes and gene mutations are shown in pink. Antimicrobial classes, AMR-related genes, and gene mutations (amino acid substitutions) are indicated next to the heatmap.

As depicted in Fig. 1, we identified a total of 35 ARGs by ResFinder and all *Salmonella* strains examined in this study possessed at least one ARG. These genes confer resistance to nine distinct classes of antimicrobials, encompassing aminoglycosides [*aac(3)-IIa*, *aac(3)-IIb*, *aac(6′)-Iaa*, *aadA1*, *aadA2*, *aadA2b*, *aph(3′)-Ia*, *aph(3′)-Ib*, *aph(3′)-IIa*, *aph(6)-Id*, and *bleO*], β-lactams (*bla*_CMY-2_, *bla*_CTX-M-14_, *bla*_CTX-M-55_, *bla*_OXA-1_, and *bla*_TEM-1B_), colistin (polymyxin E) and polymyxin B (*mcr-3.1*), fosfomycin (*fosA7*), phenicols (*catA1*, *catA2*, *catB3*, *cmlA1*, and *floR*), quinolones [*aac(6′)-Ib-cr*, *oqxAB*, *qnrB19*, *qnrD1*, and *qnrS1*], sulfonamides (*sul2* and *sul3*), tetracycline [*tet*(A), *tet*(B), and *tet*(M)], and trimethoprim (*dfrA12* and *dfrA14*).

The most prevalent gene across all isolates was *aac(6′)-Iaa*. 48.3% of study strains carried *bla*_TEM-1B_ (*n* = 14/29) and more than half harbored *tet*(A) (55.2%, *n* = 16/29). Among plasmid-mediated quinolone resistance genes (PMQRs), *qnrB19, qnrD1, qnrS1, aac(6′)-Ib-cr*, and *oqxAB* were identified in 3, 3, 18, 4, and 2 strains, respectively. Notably, one strain (*S*. Schwarzengrund strain MU18) carried 20 ARGs that included *mcr-3.1*, which contributes to colistin resistance. Strains from the same canal and year tended to exhibit similar patterns of ARGs.

Through *in silico* analysis, a total of 30 chromosomal-mediated gene mutations (= nonsynonymous mutations) in 7 genes (*acrB*, *gyrA*, *gyrB*, *parC*, *parE*, *pmrA*, and *pmrB*) were identified by ResFinder as illustrated in Fig. 1. The most prevalent mutation was in ParC and the amino acid substitution from threonine to serine at amino acid position 57 (T57S) was seen in 28 out of 29 strains (96.5%). Two amino acid substitutions at positions 28 (from phenylalanine to leucine: F28L) and 40 (from leucine to proline: L40P) were also detected in 27 out of 29 strains in AcrB (93.1%). In addition to the substitutions, several well-known mutations associated with quinolone resistance were also detected at positions 83 [from serine to phenylalanine or tyrosine: S83F (20.7%, *n* = 6/29) or S83Y (6.9%, *n* = 2/29)] and 87 [from aspartic acid to glycine: D87G (3.4%, *n* = 1/29)] of GyrA as well as at position 80 of ParC [from serine to arginine: S80R (3.4%, *n* = 1/29)] and at position 458 of ParE [from serine to proline: S458P (3.4%, *n* = 1/29)]. Among the 29 strains, 8 strains carried more than 1 amino acid substitution associated with quinolone resistance, and strain MU18 harbored 5 amino acid substitutions in GyrA, ParC, and ParE. Taken together, 22 of the 29 *Salmonella* strains (75.9%) harbored ARGs and chromosomal-mediated gene mutations across the resistance to more than 3 antimicrobial classes, classifying them as MDR strains (21).

Twenty-one *Salmonella* strains from Bangkok canal harbored both PMQR genes and chromosomal gene mutation linked to fluoroquinolones resistance. There were eight patterns of combination as follows: [1] *qnrS1* and ParC T57S (34%, *n* = 10/29), [2] *aac(6′)-Ib-cr, qnrB19, qnrS1,* GyrA S83Y, and ParC T57S (6.9%, *n* = 2/29), [3] *aac(6′)-Ib-cr, qnrS1,* GyrA S83F, and ParC T57S (6.9%, *n* = 2/29), [4] *qnrD1* and ParC T57S (6.9%, *n* = 2/29), [5] *qnrD1*, GyrA S83F*,* and ParC T57S (3.4%, *n* = 1/29), [6] *qnrB19*, GyrA S83F, and ParC T57S (3.4%, *n* = 1/29), [7] *qnrS1*, GyrA S83F-D87G, ParC T57S-S80R, and ParE S458P (3.4%, *n* = 1/29), and [8] *oqxAB*, *qnrS1*, and ParC T57S (6.9%, *n* = 2/29).

### Virulence factors in *Salmonella* strains from Bangkok canal water

**FIG 2:**
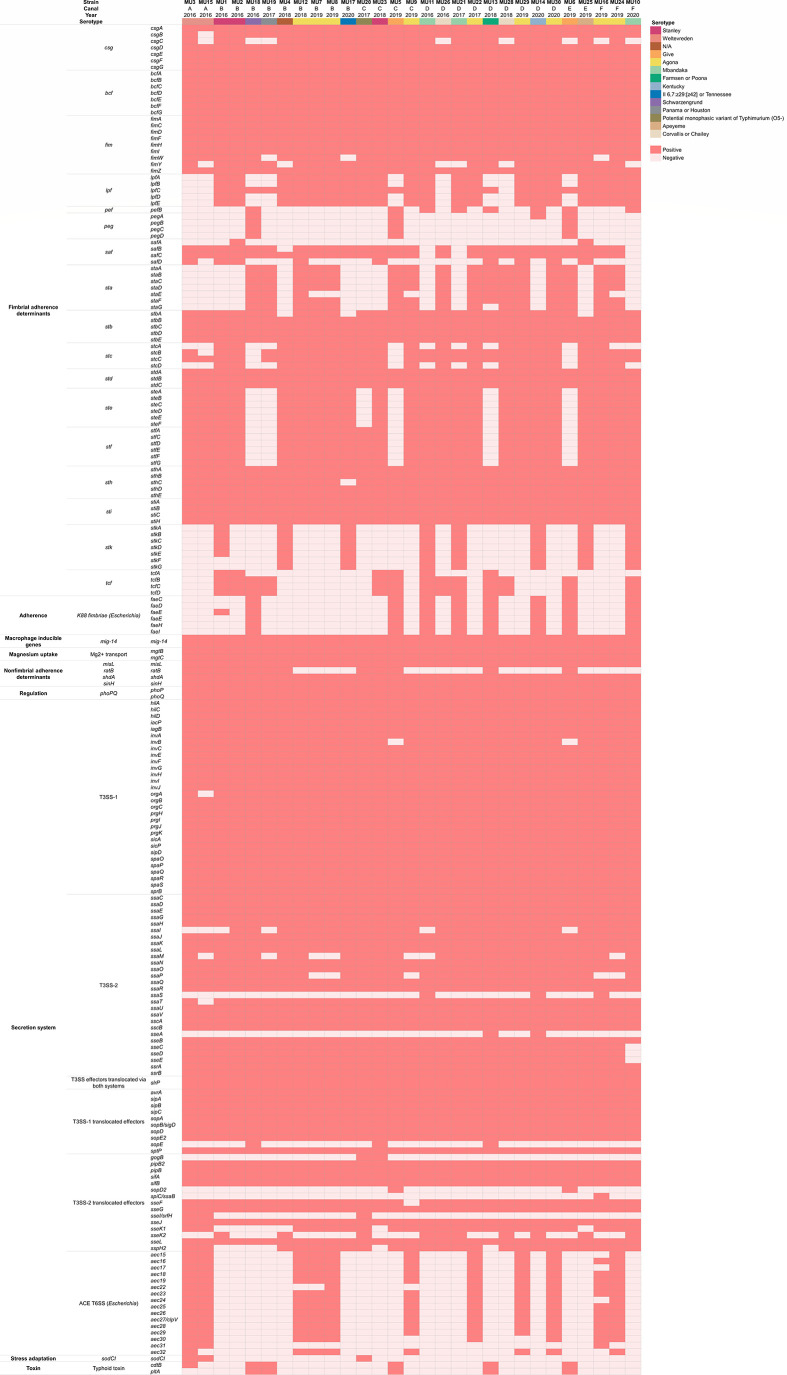
Heatmap of virulence factors in 29 *Salmonella* spp. isolated from Bangkok canal water. Present and absent genes are shown in red and pink, respectively. Virulence factors and the virulence category labels are indicated next to the heatmap.

In total, 206 virulence factors were detected as shown in Fig. 2. These virulence factors can be categorized into nine classes including fimbrial adherence determinants (*csg, bcf, fim, lpf, pef, peg, saf, sta, stb, stc, std, ste, stf, sth, sti, stk,* and *tcf*), nonfimbrial adherence determinants (*misL, ratB, shdA*, and *sinH*), adherence (K88 fimbriae), macrophage inducible genes (*mig-14*), magnesium uptake (Mg^2+^ transporter), regulation (*phoPQ*), secretion system (T3SS-1, T3SS-2, T3SS effectors translocated via both systems, T3SS-1 translocated effectors, and T3SS-2 translocated effectors), stress adaptation (*sodCI*), and toxin (typhoid toxin).

Among 206 virulence factors, 107 genes were present in all *Salmonella* genomes. The invasion gene (*invA*) and colonization gene (*fimA*) were detected in all isolates. All study strains also identified the virulence gene associated with antimicrobial peptide resistance (*mig-14*). The complete *csg* cassette was found in 21 strains. Toxin encoding genes were present in a small number of isolates. Both *cdtB* and *pltA* genes were found in five isolates from serotypes Give (strains MU5 and MU6), Farmsen or Poona (strain MU13), Schwarzengrund (strain MU18), and Panama or Houston (strain MU19), while serotype Weltevreden (strains MU3) carried only *pltA* gene. Notably, K88 fimbriae genes were observed in serotypes Give (strains MU5, MU6), Mbandaka (strains MU10, MU11, and MU21), Farmsen or Poona (strain MU13), Kentucky (strain MU14), and Schwarzengrund (strain MU18). In contrast, ACE T6SS cassettes were detected in serotypes Weltevreden (strains MU3 and MU15), and Agona (strains MU7, MU8, MU9, MU12, MU16, MU22, MU24, MU29, and MU30).

### Plasmid replicon types in *Salmonella* strains from Bangkok canal water

The plasmid replicon types of 29 *Salmonella* strains from the Bangkok canal were summarized in Table 2. In total, 15 different plasmid replicon types were detected from study strains, with Col(pHAD28), Col3M, and IncHI2 being the most common replicon types. The strains with plasmids exhibited a range of 1–5 plasmid replicon types, in which *S*. Agona strains MU7 and MU8 carried the highest number of plasmids. Notably, *qnrD1*-carrying strains were all present with Col3M plasmid replicon type, and *qnrB19*-harboring strains were present with Col(pHAD28) plasmid replicon type. However, among the study strains, eight strains did not possess any known plasmid replicon types. The strains from the same canal and year tended to display a similar pattern of plasmid replicon types.

**TABLE 2:** Plasmid replicon types of *Salmonella* spp. isolated from Bangkok canal water

Strain	Canal	Year	Plasmid replicon
MU3	A	2016	IncFII(S), IncI1-I(Alpha)
MU15	A	2016	IncFII(S)
MU1	B	2016	Col(pHAD28), IncHI2, IncHI2A
MU2	B	2016	Col(pHAD28), IncHI2, IncHI2A
MU18	B	2016	Col(BS512), IncHI2
MU19	B	2017	Col(pHAD28), IncFIA(HI1), IncFIB(K)
MU4	B	2018	Col(pHAD28), Col3M
MU12	B	2018	IncQ1, IncX1
MU7	B	2019	Col3M, IncC, IncHI2, IncHI2A, IncQ1
MU8	B	2019	Col3M, IncC, IncHI2, IncHI2A, IncQ1
MU17	B	2020	No match
MU20	C	2017	IncQ1, p0111
MU23	C	2018	No match
MU5	C	2019	Col(pHAD28)
MU9	C	2019	Col3M, IncC
MU11	D	2016	No match
MU26	D	2016	No match
MU21	D	2017	No match
MU22	D	2017	No match
MU13	D	2018	No match
MU28	D	2018	ColpVC, IncX2
MU29	D	2019	IncX1
MU14	D	2020	IncFIB(K)
MU30	D	2020	ColpVC, IncX1
MU6	E	2019	Col(pHAD28)
MU25	E	2019	No match
MU16	F	2019	Col(pHAD28), IncC
MU24	F	2019	IncX1
MU10	F	2020	Col3M

### Phylogenetic analysis of *S*. Agona from Bangkok canal water

**FIG 3:**
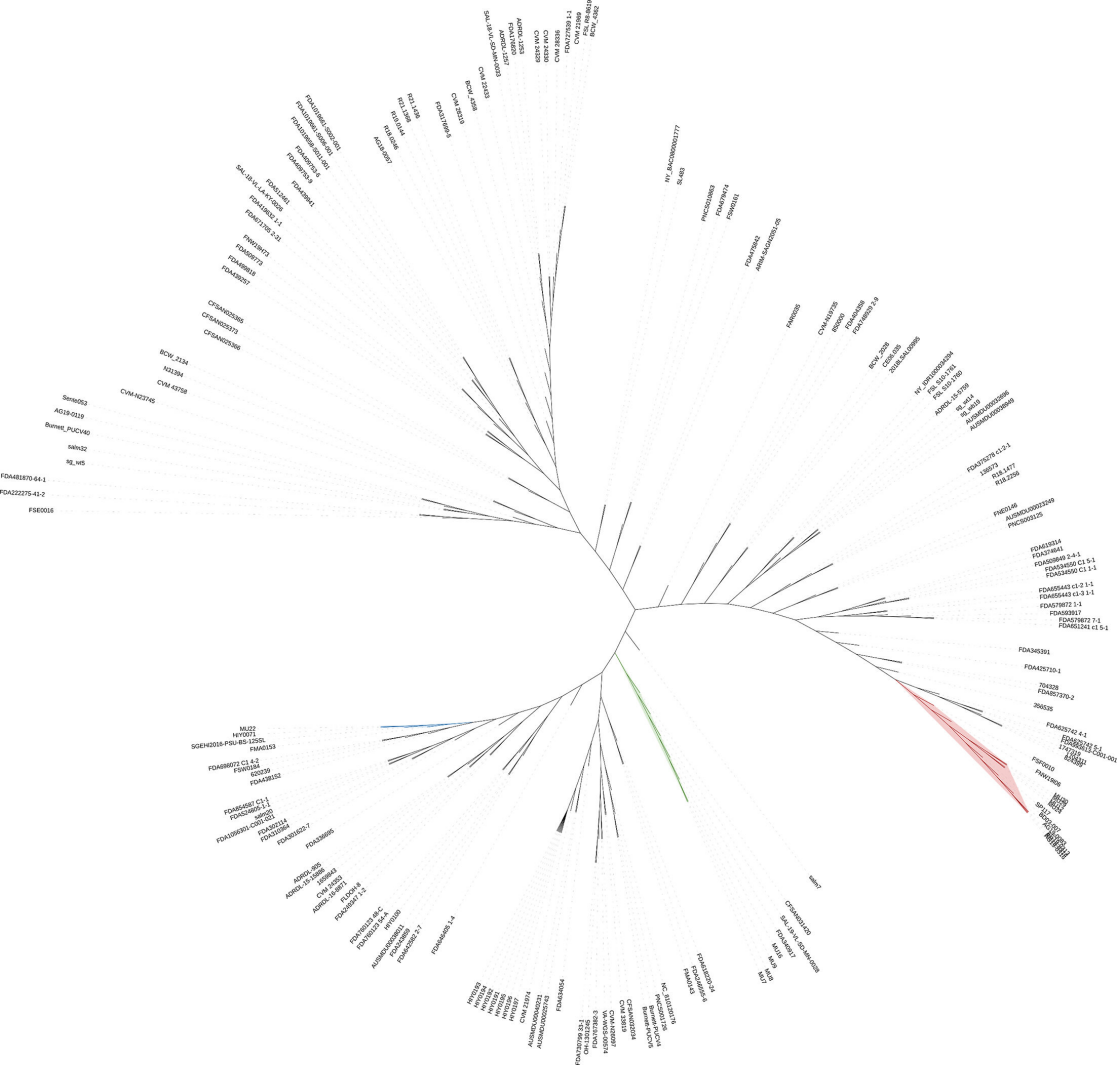
Unrooted SNP-based phylogenetic tree of 9 *S*. Agona strains from Bangkok canal water and 167 strains from the global collection. The tree illustrates the segregation of 9 *S*. Agona strains from Bangkok canal into clades 1, 2, and 3, indicated by red, green, and blue labels, respectively.

**FIG 4:**
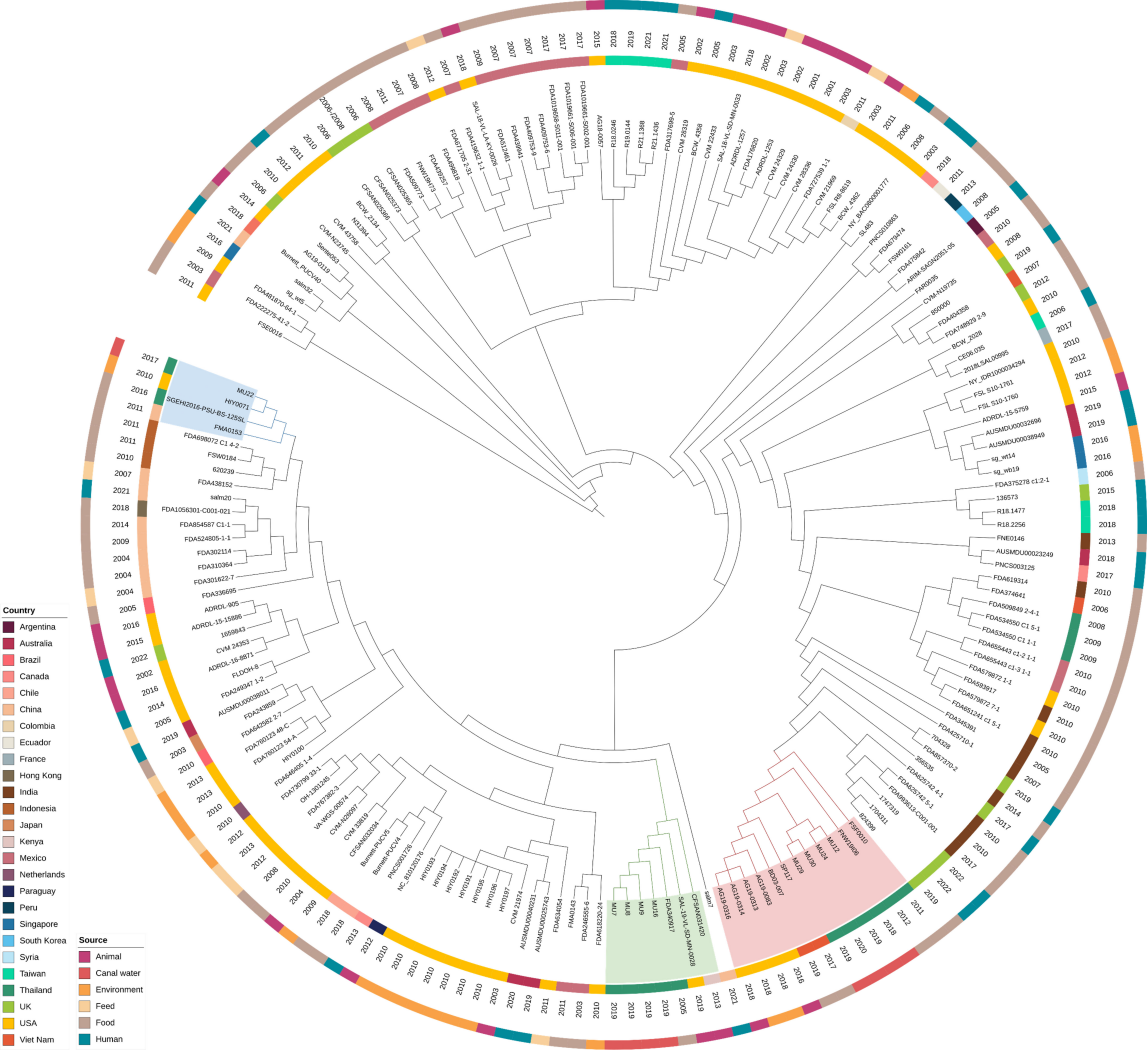
Midpoint rooted SNP-based phylogenetic tree of 9 *S*. Agona strains from Bangkok canal water and 167 strains from the global collection, with *S*. Agona strain MU9 serving as the reference point. The classification based on the isolates’ source and country is represented by different colors, following the figure’s scheme from the outer to the inner rings. Additionally, the year of isolation is displayed in the middle ring. The clades covering the study strains are depicted in red, green, and blue, corresponding to Fig. 3.

According to the single-nucleotide polymorphism (SNP)-based phylogenetic tree analysis, *S*. Agona strains obtained from canal water were categorized into three distinct clades (Fig. 3 and 4). Clade 1, depicted in red, consisted of strains MU12, MU24, MU29, and MU30 isolated from Canals B, D, and F between 2018 and 2020. Meanwhile, clade 2, represented in green, included strains MU7, MU8, MU9, and MU16 derived from Canals B, C, and F in 2019. Strain MU22, collected from Canal D in 2017, formed an independent clade marked in blue, distinctly separate from the others. Within clade 1, the canal water strains demonstrated relations to isolates sourced from food in Thailand (European Nucleotide Archive (ENA) accession no. SRR1105666 and SRR1202986) and Vietnam (ENA accession no. ERR5639045 and ERR8606799), as well as isolates from animals and the environment in the USA (ENA accession no. SRR10084910, SRR10084909, SRR10084866, and SRR8782613). In clade 2, the study strains were associated with animal strains from the USA (ENA accession no. SRR11101245) and Kenya (ENA accession no. SRR2534087), along with a food strain from Thailand (ENA accession no. SRR1726151). Within clade 3, a tested strain was clustered with an environmental strain from the USA (ENA accession no. SRR5680927), along with food strains from Thailand (ENA accession no. SRR12151684) and China (ENA accession no. SRR1288380).

## DISCUSSION

In this study, *S*. Agona was the most common *Salmonella* serotype isolated from Bangkok canal water. Over the past few years, *S*. Agona has been consistently placed on the list of the top 20 most frequently isolated serotypes in the United States (22) and the 10th most commonly reported serotype in the European Surveillance System (23). In China, *S*. Agona was reported among the top 10 serotypes that cause human diarrheal diseases (24). Moreover, *S*. Agona has been linked to several foodborne outbreaks worldwide in the last few decades and has been detected in a wide range of food and animal products, including infant milk formula, meat, chicken, and eggs associated with these outbreaks (23, 25). In Europe, *S*. Agona accounted for 13 outbreaks, resulting in 636 reported human cases and 12 hospitalizations with no deaths during 2007–2016 (23). Apart from human cases, *S*. Agona was reported as one of the most common serotypes isolated from pigs during the National Animal Health Monitoring Survey in the United States (26) and one of the most common causes of disease in both human and swine populations (27). These results suggest the potential causal risk of salmonellosis in Bangkok canals.

The most frequent ARGs detected in *Salmonella* strains from Bangkok canal water was *aac(6′)-Iaa*. This gene appeared consistently in previous studies across all *Salmonella* genomes, regardless of serotype, suggesting its presence in ancestral strains before serotype divergence and subsequent vertical transfer *via* chromosome replication (28). Other research explored that more than 98% of *S. enterica* genomes from the ENA contain *aac(6′)-Iaa* (29). While ubiquitously found in *Salmonella*, the presence or absence of this gene did not confer aminoglycoside resistance, suggesting it function as a cryptic gene (30, 31). Resistome Tracker, a tool for AMR exploration based on genome data from the National Center for Biotechnology Information (NCBI), showed that *aph(6)-Id* and *aph(3″)-Ib* were predominant aminoglycoside resistance genes in nontyphoidal *Salmonella* from environmental water (32).

This study identified the ParC T57S substitution as commonly detected in *Salmonella* strains from Bangkok canal water. This mutation alone likely has a low impact on quinolone resistance, providing minimal protection against ciprofloxacin, but is seemingly associated with enhanced bacterial fitness (33). Resistome Tracker data revealed GyrA D87Y as the predominant mutation in nontyphoidal *Salmonella* from environmental water, followed by GyrA S83F and S83Y (32), although GyrA D87Y was not found in the study strains. Multiple PMQRs were detected in *Salmonella* from Bangkok canal water, predominantly *qnrS1*. However, Resistome Tracker reported *qnrB19* as the most common PMQR gene in nontyphoidal *Salmonella* from environmental water (32). Previous Thai studies indicated a higher prevalence of *qnrS* than *qnrB* in *Salmonella* strains from various sources, including *S*. Enteritidis from humans, *S*. Choleraesuis from patients with systemic salmonellosis, and strains from pig farms and slaughterhouses (18, 34, 35). These findings suggest the circulation of *qnrS* in Thailand, indicating the country may act as a distribution hotspot for this gene.

A prior investigation revealed that *S*. Enteritidis strains co-carrying a PMQR gene and an amino acid substitution in GyrA, and those with double amino acid substitutions in GyrA displayed high levels of fluoroquinolone resistance (34). In the current study, 21 isolates of the examined strains carried both ARGs and chromosomal-mediated gene mutations. Notably, one strain contained *qnrS1*, double substitutions in GyrA and ParC, and single substitution in ParE. These findings are concerning as these isolates sourced from canal water could potentially confer elevated levels of fluoroquinolone resistance, posing a threat to treating bacterial infections and escalating public health concerns.

One *Salmonella* strain from Bangkok canal water possessed *mcr-3.1* gene that was associated with colistin resistance. According to Resistome Tracker, the predominant plasmid-mediated colistin resistance gene was *mcr-1.1*, while *mcr-3.1* was mainly found in nontyphoidal *Salmonella* isolates from humans (32). In Thailand, *mcr-3.1* was recently reported in *S*. Choleraesuis isolated from human blood (17). Colistin is considered a last-resort treatment for bacterial infections. The presence of the colistin resistance gene in *Salmonella* strain from environmental water is alarming and may indicate that canal water could serve as a potential reservoir for AMR gene distribution.

Several isolates harbored resistance genes against β-lactams (except carbapenemase-producing genes), quinolones, and tetracycline. Markedly, one strain carried resistance genes for nearly all drug classes, including colistin. These antimicrobial agents are extensively used in Thailand’s human and veterinary medicine (36). The incongruous antimicrobial usage may exert selective pressure, contributing to the widespread distribution of AMR genes. These findings underline that Bangkok canals could serve as passive vectors facilitating the further spread of MDR *Salmonella*. Addressing AMR concerns in Thailand necessitates stringent regulations on antimicrobial usage and comprehensive surveillance on a large scale. Additionally, given that canals receive water from wastewater treatment plants, improving wastewater treatment systems could help tackle the AMR issue. Currently, Bangkok’s wastewater treatment plants utilize activated sludge systems, which may not be efficient enough to remove ARGs (37). A previous study demonstrated that the membrane bioreactor was more efficient at removing ARGs than the conventional activated sludge system (38). Thus, the integrated units maximizing the advantages of different technologies, such as the combination of membrane filtration and activated sludge processes, might be a promising strategy. In addition, the local authorities might consider other alternative treatment methods, such as constructed wetlands and tridimensional eco-biological reactors (37).

The finding of invasion, adhesion, and survival genes indicates that the strains from canal water had the potential to colonize and infect humans. Additionally, the detection of K88 fimbriae adhesin and ACE T6SS cassette suggests the probable capacity of several strains from canal water to acquire new genes and functions by horizontal gene transfer. The *mig-14* gene, found in all study strains, facilitates *Salmonella* survival by blocking antimicrobial peptides (39). Alarmingly, a few *Salmonella* strains from canal water harbored genes associated with typhoid toxins like *cdtB* and *pltA*. The virulence gene identification of *Salmonella* strains assists in depicting their pathogenic potential and comprehending their occurrence and persistence in the environment.

Eight study strains did not contain any known plasmid replicon types, while some of them carried plasmid-mediated resistance genes. This may be due to the tool used in the present study is designed to detect replicons sharing a minimum of 80% nucleotide identity with the cataloged types (40). Plasmid diversity beyond this scope might be missed. Another factor to consider is the ongoing rearrangements and mutations in plasmids that potentially alter typing regions, leading to unclassifiable new types (41). Further investigation using long-read sequencing is needed for comprehensive plasmid insights in *Salmonella* from Bangkok canal water.

Phylogenetic analysis of *S*. Agona revealed that the strains from Bangkok canal water segregated into three clades. Each clade shared a common feature: the strains from canal water in this study were grouped with strains derived from food in Thailand. These findings suggest that *S*. Agona might have been circulating between environmental water and food sources in Thailand. Nonetheless, genomic data of *S*. Agona isolated from humans, animals, and environments in Thailand available in public databases are limited due to the lack of comprehensive surveillance and the infrequent use of WGS in both human and animal hospitals, slaughterhouses, water treatment plants, and other related sectors. Thus, additional genomic information is needed to establish a comprehensive genetic relationship between environmentally isolated *Salmonella* and clinically obtained *Salmonella* and understand its transmission through the One Health perspective. Additionally, the clustering of *S*. Agona from Bangkok canals with strains from foreign countries suggests the possible introduction of antimicrobial-resistant bacteria to Thailand or vice versa through international transportation. The observed relatedness among MDR *Salmonella* strains from canal water in Thailand and other countries emphasizes that MDR *Salmonella* is not solely a national but rather a global concern. This ultimately underscores the necessity for sustainable interventions to address AMR issues and safeguard public health on a global scale.

## MATERIALS AND METHODS

### Bacterial strains

**FIG 5:**
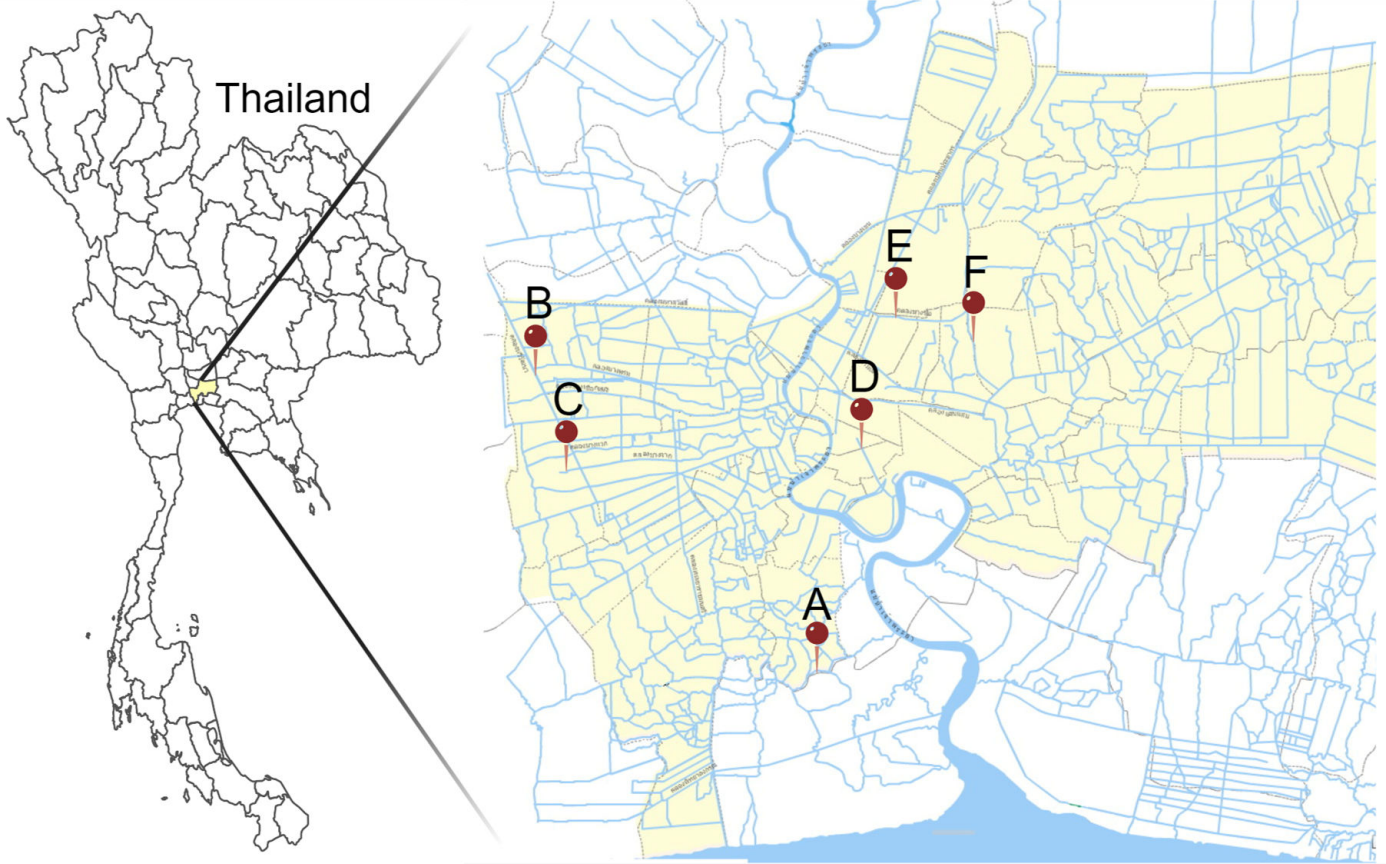
The map displays the sampling sites of six canals in Bangkok, Thailand. The yellow area denotes Bangkok, while the red pins indicate the locations of the canals (A–F). The figure was modified from reference 44 with permission and created with MapChart (https://www.mapchart.net/) and BioRender (https://BioRender.com).

Thirty *Salmonella* strains were selected based on the difference in collecting years, sampling canals, and the presence of PMQR genes (Table 3) from the *Salmonella* isolate collection of the Department of Microbiology, Faculty of Public Health, Mahidol University. These strains were obtained from water samples taken from six different canals located in Bangkok, Thailand (Fig. 5). All six canals are situated in different land-use zones as follows: Canal A in a low-density residential zone, Canal B in an agricultural zone, Canal C in a low-density residential zone, Canal D in a high-density residential zone, Canal E in a low to moderate-density residential zone, and Canal F in a moderate to high-density residential zone (42). The samples were collected across a 5-year period, from 2016 to 2020, and *Salmonella* spp. was detected in 92.2% (*n* = 307/333) of the canal water samples (J. Toyting, N. Supha, Y. Thongpanich, J. Thapa, C. Nakajima, Y. Suzuki, and F. Utrarachkij, submitted for publication). Initially, water samples were collected following the WHO’s guideline (43). The canal water samples underwent pre-enrichment by combining 50 mL of water with an equal volume of buffer peptone water. This mixture was then incubated at 37°C for 24 hours. The enriched samples were subsequently subjected to selective enrichment by being dropped (200 µL/drop, 4 drops/plate) onto Modified Semi-Solid Rappaport-Vassiliadis (MSRV) agar (Becton, Dickinson and Company, Franklin Lakes, NJ) and incubated overnight at 42°C. Subsequently, the swarming edge on the MSRV agar was selected and streaked onto Xylose Lysine Deoxycholate (XLD) agar (Becton, Dickinson and Company) and incubated for 24 hours at 37°C. Next, three presumptive *Salmonella* spp. colonies on the XLD agar were chosen for biochemical identification using Triple Sugar Iron Agar and Motility Indole Lysine Medium. Once confirmed biochemically, the isolated *Salmonella* spp. strains were preserved in Luria-Bertani (LB) broth (Becton, Dickinson and Company) supplemented with 30% glycerol and stored at −80°C for subsequent analysis. The detection of PMQR genes was performed in the previous study (J. Toyting, N. Supha, Y. Thongpanich, J. Thapa, C. Nakajima, Y. Suzuki, and F. Utrarachkij, submitted for publication), and the isolates carried more than one PMQR genes were primarily included in this study. In addition, Canal B, located in an agricultural zone, and Canal D, situated in a high-density residential area, had a high prevalence of PMQR genes. Consequently, the isolates from these canals were more frequently selected for the present study. Identification of bacterial species was performed by KmerFinder 3.2, as described in the following section, *In silico species confirmation*.

**TABLE 3:** *Salmonella* strains isolated from Bangkok canal water included in this study

Strain	Canal	Year	PMQR genes							
			*qnrA*	*qnrB*	*qnrC*	*qnrD*	*qnrS*	*aac(6′)-Ib-cr*	*qepA*	*oqxAB*
MU3	A	2016	-	-	-	-	-	-	-	-
MU15	A	2016	-	-	-	-	-	-	-	-
MU1	B	2016	-	-	-	-	+	-	-	+
MU2	B	2016	-	-	-	-	+	-	-	+
MU18	B	2016	-	-	-	-	+	-	-	-
MU19	B	2017	-	-	-	-	-	-	-	-
MU4	B	2018	-	-	-	+	-	-	-	-
MU12	B	2018	-	-	-	-	+	-	-	-
MU7	B	2019	-	-	-	-	+	+	-	-
MU8	B	2019	-	-	-	-	+	+	-	-
MU17	B	2020	-	-	-	-	-	-	-	-
MU20	C	2017	-	-	-	-	+	-	-	-
MU23	C	2018	-	-	-	-	-	-	-	-
MU5	C	2019	-	+	-	-	+	+	-	-
MU9	C	2019	-	-	-	+	-	-	-	-
MU11	D	2016	-	-	-	-	+	-	-	-
MU26	D	2016	-	-	-	-	+	-	-	-
MU21	D	2017	-	-	-	-	+	-	-	-
MU22	D	2017	-	-	-	-	+	-	-	-
MU27	D	2017	-	-	-	-	+	-	-	-
MU13	D	2018	-	-	-	-	-	-	-	-
MU28	D	2018	-	-	-	-	+	-	-	-
MU29	D	2019	-	-	-	-	+	-	-	-
MU14	D	2020	-	-	-	-	+	-	-	-
MU30	D	2020	-	-	-	-	+	-	-	-
MU6	E	2019	-	+	-	-	+	+	-	-
MU25	E	2019	-	-	-	-	-	-	-	-
MU16	F	2019	-	+	-	-	-	-	-	-
MU24	F	2019	-	-	-	-	+	-	-	-
MU10	F	2020	-	-	-	+	-	-	-	-

### DNA extraction and WGS

The bacterial genomic DNA of selected strains was extracted using Qiagen’s QIAamp DNA Mini Kit (Qiagen, Hilden, Germany) according to the manufacturer’s instructions. DNA quality and concentration were determined using NanoDrop spectrophotometer and Qubit fluorometer. A total of 1  ng of genomic DNA from each isolate was used to construct sequencing libraries using Nextera XT DNA Library Preparation Kit (Illumina Inc., San Diego, CA) following the manufacturer’s instructions. Bioanalyzer (Agilent Technologies) was used to check the library size. Selected strains were sequenced as multiplexed libraries on the Illumina MiSeq platform operated per the manufacturer’s instructions for 600 cycles to produce paired-end reads of 300  bp in length.

### Genome assembly and quality checking

The whole genome sequences of 30 *Salmonella* strains were assembled and checked for quality with FoodQCPipeline (https://bitbucket.org/genomicepidemiology/foodqcpipeline/src/master/) (45). Briefly, raw reads were pre-checked the quality with FastQC version 0.11.5 (46) and trimmed away low-quality sequences as well as sequencing adaptors with bbduk2 (part of BBtools version 36.49, https://jgi.doe.gov/data-and-tools/software-tools/bbtools/) based on an internal database. The trimmed raw reads were post-checked for quality with the FastQC version 0.11.5 (46). All QC-passed reads were according to: [1] length of reads was longer or equal to 50 bp, [2] phred score per base was higher or equal to 20 and, [3] adaptors were filtered away. The QC-passed reads were *de novo* assembled with SPAdes version 3.11.0 (47). Assemblies were assessed for quality by using Quast version 4.5 (48). The assemblies were assessed as follows: [1] number of contigs was fewer than 500 (each contig was greater than 500 bp) with an N50 value is preferably larger than 30,000 bp, [2] total number of base pairs was approximately 5,000,000 as size of *Salmonella* genome, [3] base coverage was higher or equal to 30× (base pairs sequenced)/(total base pairs after assembling), and [4] phred score was higher or equal to 20 calculated as a part of trimming process.

### *In silico* species confirmation, serotyping, sequence typing, AMR determinant and virulence factor detection, and plasmid replicon typing

Species confirmation of each stain was performed using the k-mer algorithm in KmerFinder 3.2 (https://cge.food.dtu.dk/services/KmerFinder/) (49–51). The strains that were not identified as *Salmonella enterica* subsp. *enterica* were omitted from further analysis. SeqSero2 was used to predict serotypes based on the sequences of the O (*wzx* and *wzy* genes) and H antigens (*fliC* and *fljB* genes) (52). Multilocus sequence typing (MLST) was performed using WGS data on the sequences of seven housekeeping genes (*aroC*, *dnaN*, *hemD*, *hisD*, *purE*, *sucA*, and *thrA*) to determine STs of each strain (https://cge.food.dtu.dk/services/MLST/) (53–56). AMR determinants both acquire resistance genes and chromosomal-mediated gene mutations were detected from assembled genomes by ResFinder (https://cge.food.dtu.dk/services/ResFinder/) (56–58). The pipeline was run with the default parameters with a similarity of 95% and an alignment coverage of 60%. Virulence factors were identified by VFanalyzer based on the information in the virulence factor database (VFDB) (http://www.mgc.ac.cn/cgi-bin/VFs/v5/main.cgi?func=VFanalyzer) (59). Plasmid replicon type was classified by PlasmidFinder (https://cge.food.dtu.dk/services/PlasmidFinder/) (40, 56). The pipeline was executed using default settings, including a 95% similarity and 60% alignment coverage threshold.

### Phylogenetic analysis of *S*. Agona

The phylogenetic analysis was conducted on the predominant serotype in this study, *S*. Agona. Nine assembled genomes of *S*. Agona from this study (strain MU7, MU8, MU9, MU12, MU16, MU22, MU24, MU29, and MU30) and 167 genomes from the global collection (Table S1) were used to determine genetic relatedness using Call SNPs & Infer Phylogeny (CSI Phylogeny) version 1.4 (60). *S*. Agona strain MU9 was used as a reference strain. The SNPs were determined based on default parameters as criteria: [1] a minimum depth of 10 reads at SNP positions, [2] a minimum relative depth of 10% at SNP positions, [3] a minimum distance of 10 bp between SNPs, [4] a minimum SNP quality of 30, [5] a minimum read mapping quality of 25 and [6] and minimum Z-score of 1.96. The concatenated SNPs were used to construct a maximum-likelihood tree using FastTree version 2.1.11 (61). The tree (Fig. 3 and 4) was visualized with iTOL (62).

## Data Availability

The raw sequences of 30 *Salmonella* strains from canal water samples were deposited in the NCBI Sequence Read Archive under the Bioproject number PRJNA1079544.

## SUPPLEMENTAL MATERIAL

Table S1 spectrum.04216-23-s0001.xlsx *S*. Agona strains from the global collection included in the phylogenetic analysis.

ASM does not own the copyrights to Supplemental Material that may be linked to, or accessed through, an article. The authors have granted ASM a non-exclusive, world-wide license to publish the Supplemental Material files. Please contact the corresponding author directly for reuse.

## References

[1] Amarasiri M, Sano D, Suzuki S. 2020. Understanding human health risks caused by antibiotic resistant bacteria (ARB) and antibiotic resistance genes (ARG) in water environments: current knowledge and questions to be answered. Crit Rev Environ Sci Technol 50:2016–2059. doi:10.1080/10643389.2019.1692611

[2] Singh AK, Kaur R, Verma S, Singh S. 2022. Antimicrobials and antibiotic resistance genes in water bodies: pollution, risk, and control. Front Environ Sci 10. doi:10.3389/fenvs.2022.830861

[3] Stanton IC, Bethel A, Leonard AFC, Gaze WH, Garside R. 2022. Existing evidence on antibiotic resistance exposure and transmission to humans from the environment: a systematic map. Environ Evid 11:8. doi:10.1186/s13750-022-00262-2 35308196 PMC8917330

[4] Larsson DGJ, Andremont A, Bengtsson-Palme J, Brandt KK, de Roda Husman AM, Fagerstedt P, Fick J, Flach C-F, Gaze WH, Kuroda M, et al.. 2018. Critical knowledge gaps and research needs related to the environmental dimensions of antibiotic resistance. Environ Int 117:132–138. doi:10.1016/j.envint.2018.04.041 29747082

[5] Huijbers PMC, Flach CF, Larsson DGJ. 2019. A conceptual framework for the environmental surveillance of antibiotics and antibiotic resistance. Environ Int 130:104880. doi:10.1016/j.envint.2019.05.074 31220750

[6] Nappier SP, Liguori K, Ichida AM, Stewart JR, Jones KR. 2020. Antibiotic resistance in recreational waters: state of the science. Int J Environ Res Public Health 17:8034. doi:10.3390/ijerph17218034 33142796 PMC7663426

[7] World Health Organization, Regional Office for South-East Asia. 2016. Burden of foodborne diseases in the South-East Asia region. World Health Organization, Regional Office for South-East Asia, New Delhi, India.

[8] Collier SA, Deng L, Adam EA, Benedict KM, Beshearse EM, Blackstock AJ, Bruce BB, Derado G, Edens C, Fullerton KE, Gargano JW, Geissler AL, Hall AJ, Havelaar AH, Hill VR, Hoekstra RM, Reddy SC, Scallan E, Stokes EK, Yoder JS, Beach MJ. 2021. Estimate of burden and direct healthcare cost of infectious waterborne disease in the United States. Emerg Infect Dis 27:140–149. doi:10.3201/eid2701.190676 33350905 PMC7774540

[9] Ibarra JA, Steele-Mortimer O. 2009. Salmonella--the ultimate insider. Salmonella virulence factors that modulate intracellular survival. Cell Microbiol 11:1579–1586. doi:10.1111/j.1462-5822.2009.01368.x 19775254 PMC2774479

[10] Kuhle V, Hensel M. 2004. Cellular microbiology of intracellular Salmonella enterica: functions of the type III secretion system encoded by Salmonella pathogenicity island 2. Cell Mol Life Sci 61:2812–2826. doi:10.1007/s00018-004-4248-z 15558211 PMC11924503

[11] Kadykalo S, Thomas J, Parmley EJ, Pintar K, Fleury M. 2020. Antimicrobial resistance of Salmonella and generic Escherichia coli isolated from surface water samples used for recreation and a source of drinking water in southwestern Ontario, Canada. Zoonoses Public Health 67:566–575. doi:10.1111/zph.12720 32511870

[12] Burjaq SZ, Abu-Romman SM. 2020. Prevalence and antimicrobial resistance of Salmonella spp. from irrigation water in two major sources in Jordan. Curr Microbiol 77:3760–3766. doi:10.1007/s00284-020-02178-x 32918569

[13] Li B, Vellidis G, Liu H, Jay-Russell M, Zhao S, Hu Z, Wright A, Elkins CA. 2014. Diversity and antimicrobial resistance of Salmonella enterica isolates from surface water in Southeastern United States. Appl Environ Microbiol 80:6355–6365. doi:10.1128/AEM.02063-14 25107969 PMC4178646

[14] Lundborg CS, Tamhankar AJ. 2017. Antibiotic residues in the environment of South East Asia. BMJ 358:j2440. doi:10.1136/bmj.j2440 28874355 PMC5598253

[15] Hengkrawit K, Tangjade C. 2022. Prevalence and trends in antimicrobial susceptibility patterns of multi-drug-resistance non-typhoidal Salmonella in Central Thailand, 2012–2019. Infect Drug Resist 15:1305–1315. doi:10.2147/IDR.S355213 35378891 PMC8976529

[16] Paveenkittiporn W, Kamjumphol W, Kerdsin A. 2021. Draft genome sequence of invasive Salmonella enterica serovar Cannstatt harboring mcr-1.1, isolated from a fatal sepsis case. Microbiol Resour Announc 10:e01270-20. doi:10.1128/MRA.01270-20 33632863 PMC7909088

[17] Oransathid W, Sukhchat P, Margulieux K, Wongpatcharamongkol N, Kormanee R, Pimsawat T, Preston L, Corey B, Vesely B, Waters N, Demons S, Lurchachaiwong W. 2022. First report: colistin resistance gene mcr-3.1 in Salmonella enterica serotype choleraesuis isolated from human blood sample from Thailand. Microb Drug Resist 28:102–105. doi:10.1089/mdr.2020.0553 34242096

[18] Eiamsam-Ang T, Tadee P, Pascoe B, Patchanee P. 2022. Genome-based analysis of infrequent Salmonella serotypes through the Thai pork production chain. Front Microbiol 13:968695. doi:10.3389/fmicb.2022.968695 36090074 PMC9453559

[19] Ong KH, Aung KT, Chan SCM, Chen SL, Ng LC, Vongkamjan K. 2021. Whole-genome sequencing analysis of Salmonella isolates from poultry farms, a slaughterhouse, and retail stalls in Thailand. Microbiol Resour Announc 10:e01063-20. doi:10.1128/MRA.01063-20 33986103 PMC8142589

[20] Jiarakul D. 2015. Khlong: Bangkok canals revitalization PhD thesis, Silpakorn University, Bangkok, Thailand

[21] Magiorakos AP, Srinivasan A, Carey RB, Carmeli Y, Falagas ME, Giske CG, Harbarth S, Hindler JF, Kahlmeter G, Olsson-Liljequist B, Paterson DL, Rice LB, Stelling J, Struelens MJ, Vatopoulos A, Weber JT, Monnet DL. 2012. Multidrug-resistant, extensively drug-resistant and pandrug-resistant bacteria: an international expert proposal for interim standard definitions for acquired resistance. Clin Microbiol Infect 18:268–281. doi:10.1111/j.1469-0691.2011.03570.x 21793988

[22] Ferrari RG, Rosario DKA, Cunha-Neto A, Mano SB, Figueiredo EES, Conte-Junior CA. 2019. Worldwide epidemiology of Salmonella serovars in animal-based foods: a meta-analysis. Appl Environ Microbiol 85:e00591-19. doi:10.1128/AEM.00591-19 31053586 PMC6606869

[23] European Food Safety Authority and European Centre for Disease Prevention and Control (EFSA and ECDC). 2018. Multi-country outbreak of Salmonella Agona infections possibly linked to ready-to-eat food. EFSA Supp Publ 15:1465E. doi:10.2903/sp.efsa.2018.EN-1465

[24] Kuang D, Zhang J, Meng J, Yang X, Jin H, Shi W, Luo K, Tao Y, Pan H, Xu X, Ren T. 2014. Antimicrobial susceptibility and molecular typing of Salmonella Agona isolated from humans and other sources. Foodborne Pathog Dis 11:844–849. doi:10.1089/fpd.2014.1776 25361176

[25] Siddique A, Ullah N, Ali A, Patel A, Moore T, Kenney SM, Ganda E, Rahman A. 2022. Draft genome sequences of 25 Salmonella enterica serovar Agona strains isolated from poultry and associated food products harbouring multiple antibiotic resistance genes. J Glob Antimicrob Resist 29:131–135. doi:10.1016/j.jgar.2022.02.013 35227945

[26] Haley CA, Dargatz DA, Bush EJ, Erdman MM, Fedorka-Cray PJ. 2012. Salmonella prevalence and antimicrobial susceptibility from the national animal health monitoring system swine 2000 and 2006 studies. J Food Prot 75:428–436. doi:10.4315/0362-028X.JFP-11-363 22410214

[27] Iowa State University College of Veterinary Medicine Department of Veterinary Diagnostic & Production Animal Medicine. Salmonellosis. Available from: https://vetmed.iastate.edu/ vdpam/FSVD/swine/index-diseases/salmonellosis. Retrieved 24 Feb 2024.

[28] Liao J, Orsi RH, Carroll LM, Kovac J, Ou H, Zhang H, Wiedmann M. 2019. Serotype-specific evolutionary patterns of antimicrobial-resistant Salmonella enterica. BMC Evol Biol 19:132. doi:10.1186/s12862-019-1457-5 31226931 PMC6588947

[29] Nuanmuang N, Leekitcharoenphon P, Njage PMK, Gmeiner A, Aarestrup FM. 2023. An overview of antimicrobial resistance profiles of publicly available Salmonella genomes with sufficient quality and metadata. Foodborne Pathog Dis 20:405–413. doi:10.1089/fpd.2022.0080 37540138 PMC10510693

[30] Feldgarden M, Brover V, Gonzalez-Escalona N, Frye JG, Haendiges J, Haft DH, Hoffmann M, Pettengill JB, Prasad AB, Tillman GE, Tyson GH, Klimke W. 2021. AMRFinderPlus and the reference gene catalog facilitate examination of the genomic links among antimicrobial resistance, stress response, and virulence. Sci Rep 11:12728. doi:10.1038/s41598-021-91456-0 34135355 PMC8208984

[31] Magnet S, Courvalin P, Lambert T. 1999. Activation of the cryptic aac(6’)-Iy aminoglycoside resistance gene of Salmonella by a chromosomal deletion generating a transcriptional fusion. J Bacteriol 181:6650–6655. doi:10.1128/JB.181.21.6650-6655.1999 10542165 PMC94128

[32] FDA. 2023. Global resistome data. Available from: https://www.fda.gov/animal-veterinary/national-antimicrobial-resistance-monitoring-system/global-resistome-data. Retrieved 20 Nov 2023.

[33] Chang M-X, Zhang J-F, Sun Y-H, Li R-S, Lin X-L, Yang L, Webber MA, Jiang H-X. 2021. Contribution of different mechanisms to ciprofloxacin resistance in Salmonella spp. Front Microbiol 12:663731. doi:10.3389/fmicb.2021.663731 34025618 PMC8137344

[34] Utrarachkij F, Nakajima C, Changkwanyeun R, Siripanichgon K, Kongsoi S, Pornruangwong S, Changkaew K, Tsunoda R, Tamura Y, Suthienkul O, Suzuki Y. 2017. Quinolone resistance determinants of clinical Salmonella Enteritidis in Thailand. Microb Drug Resist 23:885–894. doi:10.1089/mdr.2015.0234 28437229

[35] Sriyapai P, Pulsrikarn C, Chansiri K, Nyamniyom A, Sriyapai T. 2021. Molecular characterization of cephalosporin and fluoroquinolone resistant Salmonella Choleraesuis isolated from patients with systemic salmonellosis in Thailand. Antibiotics (Basel) 10:844. doi:10.3390/antibiotics10070844 34356765 PMC8300840

[36] Health Policy and Systems Research on Antimicrobial Resistance Network. 2022. Thailand’s one health report on antimicrobial consumption and antimicrobial resistance in 2020

[37] Yu K-F, Li P, Zhang B, He Y. 2023. Technologies to tackle antimicrobial resistance during treated wastewater reuse: current advances and future prospects. Curr Opin Chem Eng 42:100951. doi:10.1016/j.coche.2023.100951

[38] Munir M, Wong K, Xagoraraki I. 2011. Release of antibiotic resistant bacteria and genes in the effluent and biosolids of five wastewater utilities in Michigan. Water Res 45:681–693. doi:10.1016/j.watres.2010.08.033 20850863

[39] Brodsky IE, Ghori N, Falkow S, Monack D. 2005. Mig-14 is an inner membrane-associated protein that promotes Salmonella typhimurium resistance to CRAMP, survival within activated macrophages and persistent infection. Mol Microbiol 55:954–972. doi:10.1111/j.1365-2958.2004.04444.x 15661016

[40] Carattoli A, Zankari E, García-Fernández A, Voldby Larsen M, Lund O, Villa L, Møller Aarestrup F, Hasman H. 2014. In silico detection and typing of plasmids using PlasmidFinder and plasmid multilocus sequence typing. Antimicrob Agents Chemother 58:3895–3903. doi:10.1128/AAC.02412-14 24777092 PMC4068535

[41] Rozwandowicz M, Brouwer MSM, Fischer J, Wagenaar JA, Gonzalez-Zorn B, Guerra B, Mevius DJ, Hordijk J. 2018. Plasmids carrying antimicrobial resistance genes in Enterobacteriaceae. J Antimicrob Chemother 73:1121–1137. doi:10.1093/jac/dkx488 29370371

[42] Department of City Planning BMA. 2015. Inspection system for comprehensive city plans and Bangkok ordinances. Available from: https://cityplangis.bangkok.go.th/bma_cpudd/cmpweb. Retrieved 24 Feb 2024.

[43] World Health Organization. 1997. Guidelines for drinking water quality. 2nd ed. Surveillance and control of community supplies.

[44] Boonpromgul P, Boon-Long Y. 2012. The design and development of sustainable cities: international and Thai perspectives on urban design in the 21st century. Faculty of Architecture and Planning, Thammasat University.

[45] CGE. 2016. FoodQCPipeline. Available from: https://bitbucket.org/genomicepidemiology/foodqcpipeline /src/master. Retrieved 08 May 2023.

[46] Babraham Bioinformatics. 2016. FastQC: a quality control tool for high throughput sequence data. Available from: https://www.bioinformatics.babraham.ac.uk/projects/fastqc. Retrieved 08 May 2023.

[47] Bankevich A, Nurk S, Antipov D, Gurevich AA, Dvorkin M, Kulikov AS, Lesin VM, Nikolenko SI, Pham S, Prjibelski AD, Pyshkin AV, Sirotkin AV, Vyahhi N, Tesler G, Alekseyev MA, Pevzner PA. 2012. SPAdes: a new genome assembly algorithm and its applications to single-cell sequencing. J Comput Biol 19:455–477. doi:10.1089/cmb.2012.0021 22506599 PMC3342519

[48] Gurevich A, Saveliev V, Vyahhi N, Tesler G. 2013. QUAST: quality assessment tool for genome assemblies. Bioinformatics 29:1072–1075. doi:10.1093/bioinformatics/btt086 23422339 PMC3624806

[49] Hasman H, Saputra D, Sicheritz-Ponten T, Lund O, Svendsen CA, Frimodt-Møller N, Aarestrup FM. 2014. Rapid whole-genome sequencing for detection and characterization of microorganisms directly from clinical samples. J Clin Microbiol 52:139–146. doi:10.1128/JCM.02452-13 24172157 PMC3911411

[50] Larsen MV, Cosentino S, Lukjancenko O, Saputra D, Rasmussen S, Hasman H, Sicheritz-Pontén T, Aarestrup FM, Ussery DW, Lund O. 2014. Benchmarking of methods for genomic taxonomy. J Clin Microbiol 52:1529–1539. doi:10.1128/JCM.02981-13 24574292 PMC3993634

[51] Clausen PTLC, Aarestrup FM, Lund O. 2018. Rapid and precise alignment of raw reads against redundant databases with KMA. BMC Bioinformatics 19:307. doi:10.1186/s12859-018-2336-6 30157759 PMC6116485

[52] Zhang S, Yin Y, Jones MB, Zhang Z, Deatherage Kaiser BL, Dinsmore BA, Fitzgerald C, Fields PI, Deng X. 2015. Salmonella serotype determination utilizing high-throughput genome sequencing data. J Clin Microbiol 53:1685–1692. doi:10.1128/JCM.00323-15 25762776 PMC4400759

[53] Larsen MV, Cosentino S, Rasmussen S, Friis C, Hasman H, Marvig RL, Jelsbak L, Sicheritz-Pontén T, Ussery DW, Aarestrup FM, Lund O. 2012. Multilocus sequence typing of total-genome-sequenced bacteria. J Clin Microbiol 50:1355–1361. doi:10.1128/JCM.06094-11 22238442 PMC3318499

[54] Bartual SG, Seifert H, Hippler C, Luzon MAD, Wisplinghoff H, Rodríguez-Valera F. 2005. Development of a multilocus sequence typing scheme for characterization of clinical isolates of Acinetobacter baumannii. J Clin Microbiol 43:4382–4390. doi:10.1128/JCM.43.9.4382-4390.2005 16145081 PMC1234098

[55] Jaureguy F, Landraud L, Passet V, Diancourt L, Frapy E, Guigon G, Carbonnelle E, Lortholary O, Clermont O, Denamur E, Picard B, Nassif X, Brisse S. 2008. Phylogenetic and genomic diversity of human bacteremic Escherichia coli strains. BMC Genomics 9:560. doi:10.1186/1471-2164-9-560 19036134 PMC2639426

[56] Camacho C, Coulouris G, Avagyan V, Ma N, Papadopoulos J, Bealer K, Madden TL. 2009. BLAST+: architecture and applications. BMC Bioinformatics 10:421. doi:10.1186/1471-2105-10-421 20003500 PMC2803857

[57] Bortolaia V, Kaas RS, Ruppe E, Roberts MC, Schwarz S, Cattoir V, Philippon A, Allesoe RL, Rebelo AR, Florensa AF, et al.. 2020. ResFinder 4.0 for predictions of phenotypes from genotypes. J Antimicrob Chemother 75:3491–3500. doi:10.1093/jac/dkaa345 32780112 PMC7662176

[58] Zankari E, Hasman H, Cosentino S, Vestergaard M, Rasmussen S, Lund O, Aarestrup FM, Larsen MV. 2012. Identification of acquired antimicrobial resistance genes. J Antimicrob Chemother 67:2640–2644. doi:10.1093/jac/dks261 22782487 PMC3468078

[59] Liu B, Zheng D, Zhou S, Chen L, Yang J. 2022. VFDB 2022: a general classification scheme for bacterial virulence factors. Nucleic Acids Res 50:D912–D917. doi:10.1093/nar/gkab1107 34850947 PMC8728188

[60] Kaas RS, Leekitcharoenphon P, Aarestrup FM, Lund O. 2014. Solving the problem of comparing whole bacterial genomes across different sequencing platforms. PLoS One 9:e104984. doi:10.1371/journal.pone.0104984 25110940 PMC4128722

[61] Price MN, Dehal PS, Arkin AP. 2010. FastTree 2 – approximately maximum-likelihood trees for large alignments. PLoS One 5:e9490. doi:10.1371/journal.pone.0009490 20224823 PMC2835736

[62] Letunic I, Bork P. 2019. Interactive tree of life (iTOL) v4: recent updates and new developments. Nucleic Acids Res 47:W256–W259. doi:10.1093/nar/gkz239 30931475 PMC6602468

